# Xenobiotic Effects of Chlorine Dioxide to *Escherichia coli* O157:H7 on Non-host Tomato Environment Revealed by Transcriptional Network Modeling: Implications to Adaptation and Selection

**DOI:** 10.3389/fmicb.2020.01122

**Published:** 2020-06-03

**Authors:** Xiaomei Shu, Manavi Singh, Naga Bhushana Rao Karampudi, David F. Bridges, Ai Kitazumi, Vivian C. H. Wu, Benildo G. De los Reyes

**Affiliations:** ^1^Department of Plant and Soil Science, Texas Tech University, Lubbock, TX, United States; ^2^Produce Safety and Microbiology Research, Western Regional Research Center, United States Department of Agriculture – Agricultural Research Service, Albany, CA, United States

**Keywords:** *Escherichia coli*, *Solanum lycopersicum*, gaseous chlorine dioxide, RNA-Seq, transcriptional regulatory network, functional co-expression modules, supra-optimal effects

## Abstract

*Escherichia coli* serotype O157:H7 is one of the major agents of pathogen outbreaks associated with fresh fruits and vegetables. Gaseous chlorine dioxide (ClO_2_) has been reported to be an effective intervention to eliminate bacterial contamination on fresh produce. Although remarkable positive effects of low doses of ClO_2_ have been reported, the genetic regulatory machinery coordinating the mechanisms of xenobiotic effects and the potential bacterial adaptation remained unclear. This study examined the temporal transcriptome profiles of *E. coli* O157:H7 during exposure to different doses of ClO_2_ in order to elucidate the genetic mechanisms underlying bacterial survival under such harsh conditions. Dosages of 1 μg, 5 μg, and 10 μg ClO_2_ per gram of tomato fruits cause different effects with dose-by-time dynamics. The first hour of exposure to 1 μg and 5 μg ClO_2_ caused only partial killing with significant growth reduction starting at the second hour, and without further significant reduction at the third hour. However, 10 μg ClO_2_ exposure led to massive bacterial cell death at 1 h with further increase in cell death at 2 and 3 h. The first hour exposure to 1 μg ClO_2_ caused activation of primary defense and survival mechanisms. However, the defense response was attenuated during the second and third hours. Upon treatment with 5 μg ClO_2_, the transcriptional networks showed massive downregulation of pathogenesis and stress response genes at the first hour of exposure, with decreasing number of differentially expressed genes at the second and third hours. In contrast, more genes were further downregulated with exposure to 10 μg ClO_2_ at the first hour, with the number of both upregulated and downregulated genes significantly decreasing at the second hour. A total of 810 genes were uniquely upregulated at the third hour at 10 μg ClO_2_, suggesting that the potency of xenobiotic effects had led to potential adaptation. This study provides important knowledge on the possible selection of target molecules for eliminating bacterial contamination on fresh produce without overlooking potential risks of adaptation.

## Introduction

Illnesses caused by the foodborne enteropathogen and Shiga toxin-producing *Escherichia coli* O157 (STEC) can be life-threatening (Nataro and Kaper, 1998; Law, 2001; Santiago et al., 2017). Most of the STEC strains identified by USDA carry toxin genes *stx1a* and *stx2a* (González-Escalona and Kase, 2019). *E. coli* serotype O157:H7 has been reported to be the source of outbreaks on fresh produce such as tomato fruits (*Solanum lycopersicum* L.) (Gomez-Aldapa et al., 2013). Additionally, it has been reported that STEC contamination of non-host tomato occurred at different developmental stages through various routes (Hwang et al., 2017; Bridges et al., 2018). Fertilizer, irrigation water, insect vectors, soil, or seed could harbor this human pathogen (Deering et al., 2015; Ocana de Jesus et al., 2018). STEC is capable of contaminating tomato with significant wounding, which provides enormous inocula that can cause cross-contamination during subsequent post-harvest processing and transportation (Yeni et al., 2016; Cai et al., 2018).

Because *E. coli* O157:H7 is one of the most dangerous serotypes threatening public health (Watanabe et al., 1996; Yang et al., 2017), different strategies such as on-farm hygiene, washing, film coating, prophage induction, and use of interventions through sodium hypochlorite (NaClO), sodium chlorite (NaClO_2_), acidified sodium benzoate (NaB), and peracetic acid (PAA) have been applied to control the bacterial population retained on the surface of fresh tomato (Cadieux et al., 2018; Chen and Zhong, 2018; Singh et al., 2018). However, effective, safe, and sustainable treatment strategies that reduce fruit contamination have been difficult to achieve possibly due in large part to the poor understanding of the molecular and genetic mechanisms involved in the responses of *E. coli* to chemical treatments. Potential acclimation, adaptation, mutation, and selection caused by chronic exposure to selective doses of intervention agents are often overlooked. Moreover, the non-host environments add another layer of stress effects that might also induce cross-protection to the bacteria (Chen and Zhong, 2018). Thus, a comprehensive understanding of the possible negative consequences caused by chronic exposure to different doses of chemical intervention is important to prevent future outbreaks caused by STEC (Bridges et al., 2018).

Gaseous chlorine dioxide (ClO_2_) is known to be an efficient chemical intervention agent with its xenobiotic effects to eliminate the hitch-hiking human pathogenic *E. coli* O157:H7 on tomato produce (Bridges et al., 2018). Our previous studies led to a hypothesis that low doses of ClO_2_ (lower than commercial processing practices) could be both an optimal and supra-optimal dose that could result in either effective bacterial growth reduction or bacterial adaptive response depending on dose × time dynamics (Bridges et al., 2018). However, new outbreaks indicated that more research needs to be conducted to ensure maximal efficiency without the possibility of forcing adaptation and selection that could cause subsequent repeated outbreaks (i.e., selection pressure). The establishment of a sustainable ClO_2_-based treatment protocol with high efficiency requires understanding of how certain serotypes of STEC respond to the potential xenobiotic effects of various doses of ClO_2_ at different exposure duration. Understanding the genetic mechanisms governing STECs defense against ClO_2_ could facilitate fine-tuned protocols for delivering an optimal intervention that reduces the potential risks of future new outbreaks.

In this study, we conducted transcriptome studies using RNA-Seq technology to reveal the genetic mechanism of *E. coli* O157:H7 in response to xenobiotic effects of different doses of ClO_2_ on its non-host tomato across a range of exposure time. By dissecting the global dynamics of transcriptome changes (Bridges et al., 2018; Santos-Zavaleta et al., 2018), we reconstructed the regulatory networks associated with the responses of *E. coli* O157:H7 to 1 μg, 5 μg, and 10 μg of ClO_2_ per gram of tomato fruits. We uncovered the potential network hubs that are likely to be critical in executing defense responses and possible adaptation. We also demonstrated that supra-optimal exposure time (i.e., the third hour) under 10 μg ClO_2_ could potentially lead to a new burst of independent defense mechanism after the killing phase during the first and second hours. This points to the probable occurrence of adaptation and selection. Our current results provide critical information for understanding the risk of developing chemical resistance in *E. coli*, hence adaptation and selection that could possibly nucleate future new outbreaks.

## Materials and Methods

### Bacterial Inocula Preparation and Inoculation

*Escherichia coli* O157:H7 (ATCC 35150) was obtained from the Pathogenic Microbiology Laboratory of the United States Department of Agriculture-Agricultural Research Service, Western Regional Research Center. The bacterial strain was cultured in tryptic soy broth (TSB; Sigma-Aldrich, United States) overnight at 37°C, centrifuged at 5000 × g for 15 min, re-suspended in 10 ml 0.1% peptone water, centrifuged for another 15 min, and re-suspended in 12 ml 0.1% peptone water.

Fresh tomato produce without post-harvest processing was obtained from Windset Farms (California). Tomato fruits without visual damage or fungal growth were washed with water and 70% ethanol, then dried in the hood. For each tomato fruit, 250 μl of *E. coli* suspension was inoculated on the surface, allowed to air dry for a few hours, placed in sterile bags, and incubated overnight at 4°C to prevent massive bacterial growth. Twelve tomato fruits were weighed and used for each ClO_2_ treatment. Three replicates consisting of 12 tomato fruits within each replicate were included for each treatment by time point combination. Tomato fruits were treated with 1, 5, and 10 μg ClO_2_ at room temperature as previously described (Bridges et al., 2018). In our preliminary studies, we found that these dosages, which are much lower than the dosages used in food industry, are ideal for this study, creating a system that causes significant bacterial growth reduction with enough surviving cells for gene expression analysis. The intent was to create a transcriptome analysis system that reflects cellular responses, rather than the noise caused by RNA degradation from massive cell death.

### Bacterial Growth Assay

After 1, 2, and 3 h of ClO_2_ exposure, each tomato fruit was rinsed with 10 ml 0.1% peptone water for 1 min for serial dilutions (10^–1^ to 10^–5^) and plated using MacConkey Sorbital Agar supplemented with Cefixime and Tellurite, layered with thin layer tryptic soy agar (TSA; Sigma-Aldrich, United States). Samples were incubated overnight at 37°C and bacterial growth (log CFU/g) was determined by comparing ClO_2_-treated samples with the control.

### RNA Purification, RNA-Seq Library Construction, Sequencing, and Data Processing

*Escherichia coli* RNA samples were isolated using a Quick-RNA Fungal/Bacterial RNA Microprep kit (Zymo Research) according to the manufacturer’s instructions. For each sample, two replicates were included to construct RNA-Seq libraries with 900× coverage per library, then sequenced with 150-bp paired-end reads on Illumina HiSeq-3000 (Genomics Core Facility, Oklahoma Medical Research Foundation, Norman, OK, United States).

RNA-Seq raw reads were processed according to previous studies (Kitazumi et al., 2018). Raw data were preprocessed with Cutadapt (version v1.9.1) to remove adapters and low-quality sequences to generate paired 100-bp reads (Martin, 2011). Subsequently, the data with at least 16 million pairs per library were mapped using Edge-Pro (version v1.3.1) to account for polycistronic gene organization (Magoc et al., 2013). Reference *E. coli* O157:H7 str. Sakai genome (GenBank: GCA_000008865.2, NCBI: ASM886v2) was used for mapping based on high map rates (∼98%) of control sample and availability of pathway annotation in KEGG (organism code ecs^1^) (Kanehisa and Goto, 2000).

### Propensity Transformation and Transcriptional Regulatory Network Analysis

Two biological replicates were included for control (*t*_0_), 1 (*t*_1_), 2 (*t*_2_), and 3 h (*t*_3_). Average reads per kilobase of transcript, per million mapped reads (RPKM) were transformed using the propensity transformation (PT) methodology with the equation shown below.

$$P\,t\,i=\ln\left(\frac{\frac{T_{i}}{\sum_{j=t_{0}}^{t_{3}}T_{i\,j}}}{\frac{\sum_{i=1}^{5192}T_{j}}{\sum_{j=t_{0}}^{t_{3}}\sum_{i=1}^{5192}T_{i\,j}}}\right)$$

Where,

*Pt_*i*_* = Propensity transformation of RPKM value of transcript *i.*

*T_*i*_* = RPKM value of transcript *i.*

*j* = Variable that iterates over datasets of *t_0_* = control, *t_1_* = 1 h, *t*_2_ = 2 h and *t*_3_ = 3 h.

*i* = Variable that iterates over the total number of transcript-encoding loci (i.e., 5,129 transcripts per dataset).

Missing data was considered as NULL and transformed to value of 0, hence not significant. The PT data for each library showed a normal distribution ranging between −n to +n, then was further fragmented into 20 quartiles based on the propensity scores. Quartile cuts resulted in 250 transcripts per quartile in all the datasets. Two quartiles per dataset representing the transcripts with the lowest and the highest propensity scores were selected for transcriptional network analysis resulting in 500 transcript-encoding loci per dataset. A total number of 1,239, 1,318, and 1,286 transcripts were selected from 1, 5, and 10 μg ClO_2_ treatments, respectively, including control, 1, 2, and 3 h in each dosage. The overlaps between the datasets resulted in less than the total number of expected 2,000 transcripts (500 × 4 datasets) because of the overlapped transcripts between time points. Two-way hierarchical clustering analysis with PT values was performed using JMP, 11 (SAS Institute Inc., Cary, NC, United States).

### Network Modeling and Module Analysis

Based on the distribution of PT values, a subset of normalized RPKM values were selected to calculate the standard Pearson Correlation Coefficient (PCC) using the Python Pandas library. The dataset was derived from the PT values without log transformation containing only positive values. The PCC for one versus all transcripts were calculated using this subset of normalized RPKM that resulted in a diagonally symmetrical matrix of 5129 × 5129 coefficients, in which the diagonal values represent the PCC of every transcript locus with itself.

Transcript-encoding loci for network modeling were selected using the propensity score followed by the PCC. Propensity-based selection was described in the previous section. A cut-off of 0.9999 was subsequently used for filtering out both positively and negatively correlated transcript loci. The selection represented gene loci that were significant according to propensity scores and their highly correlated co-upregulated, co-downregulated, or inversely co-expressed loci from the primary selection. Network module was used to plot the networks where each transcript was represented as a node, while the correlation coefficient is depicted using the connection between nodes.

### Data Deposition

RNA-Seq data generated in this study were deposited at the National Center for Biotechnology Information (NCBI) Sequence Read Archive (SRA) collection under the accession number SRR8468286-9.

## Results

### *E. coli* Growth Reduction and Recovery During ClO_2_ Xenobiotic Effects

In order to understand the impact of xenobiotic effects of different ClO_2_ dosage, and exposure time on bacterial viability and regrowth ability, and the potential impact of prolonged time to potential supra-optimal effects, we assessed the survival of *E. coli* O157:H7 on the surface of tomato produce at three different dosages of gaseous ClO_2_ (1, 5, and 10 μg per grams of ripen fruits) at room temperature. Growth rate was assessed across different durations of exposure 1, 2, and 3 h, respectively.

**FIGURE 1:**
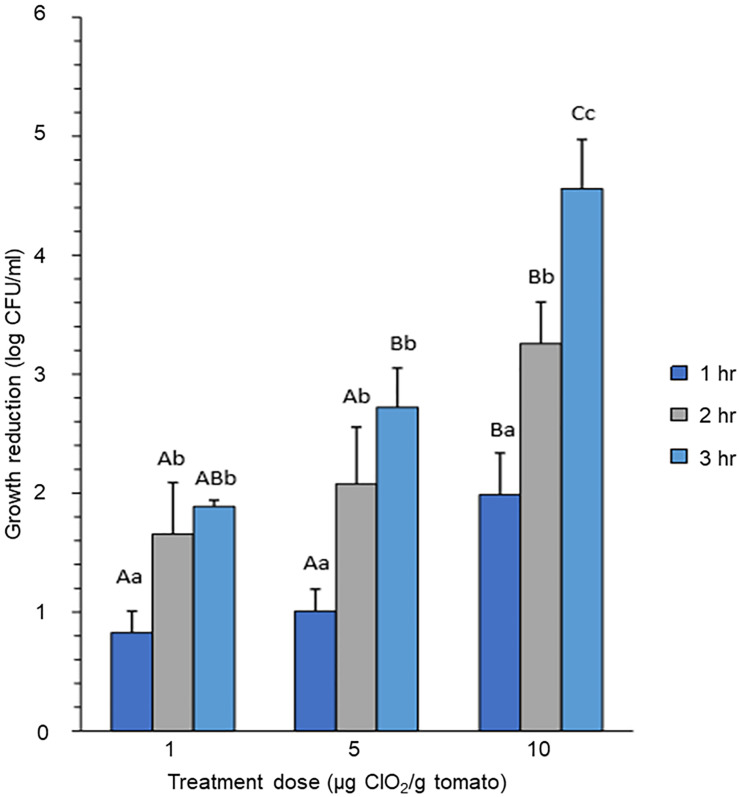
Growth reduction of *E. coli* on tomato treated with different concentrations of gaseous ClO_2_. Tomato fruits were treated with 1 μg, 5 μg, or 10 μg ClO_2_ per gram of ripe berries. Upper-case letters indicate significant difference (*P* < 0.05) caused by ClO_2_ doses at the same time point. Lower-case letters indicate significant difference (*P* < 0.05) caused by time of exposure under the same ClO_2_ dosage.

Bacterial cell count expressed as log-reduction was significantly reduced after 2 h exposure to both 1 and 5 μg ClO_2_ per gram of ripe fruits (*P* < 0.05) compared to a subtle reduction after 1 h exposure (*P* > 0.05). Although the mean cell recoveries from the tomato surface washings appeared to be further reduced at 3 h compared to 2 h exposure, this difference was not statistically significant (*P* > 0.05) (Figure 1). In contrast, *E. coli* exposed to 10 μg ClO_2_ showed significant growth reduction at the first hour (*P* < 0.05), with further dramatic decreases in growth rates at both the second (*P* < 0.05) and third hours (*P* < 0.05) (Figure 1). Comparing growth reduction under different dosages of ClO_2_ at the same exposure time, we observed that 10 μg ClO_2_ caused significant log-reduction compared with either 1 or 5 μg ClO_2_ (Figure 1). Since optimizing dosage and exposure time to the xenobiotic agent are of primary importance as a means of preserving the integrity and quality of the produce, our data implied that exposure to 10 μg ClO_2_ resulted in similar effects on bacterial killing, thus a higher dosage with shorter exposure time is adequate for optimal effect. These results further support the hypothesis that the unnecessary additional hours of exposure may not only potentially cause negative effects on the post-harvest quality of the fresh produce, but perhaps trigger adaptation and selection on the surviving sub-populations of bacteria. We hypothesized that prolonged exposure to high doses of ClO_2_ could possibly impose a potential risk of supra-optimal effects that may lead to bacterial adaptation and selection, hence possibly providing the potent inoculum for future outbreaks.

### Overall Trends of *E. coli* Transcriptomic Changes in Response to ClO_2_ Exposure

**FIGURE 2:**
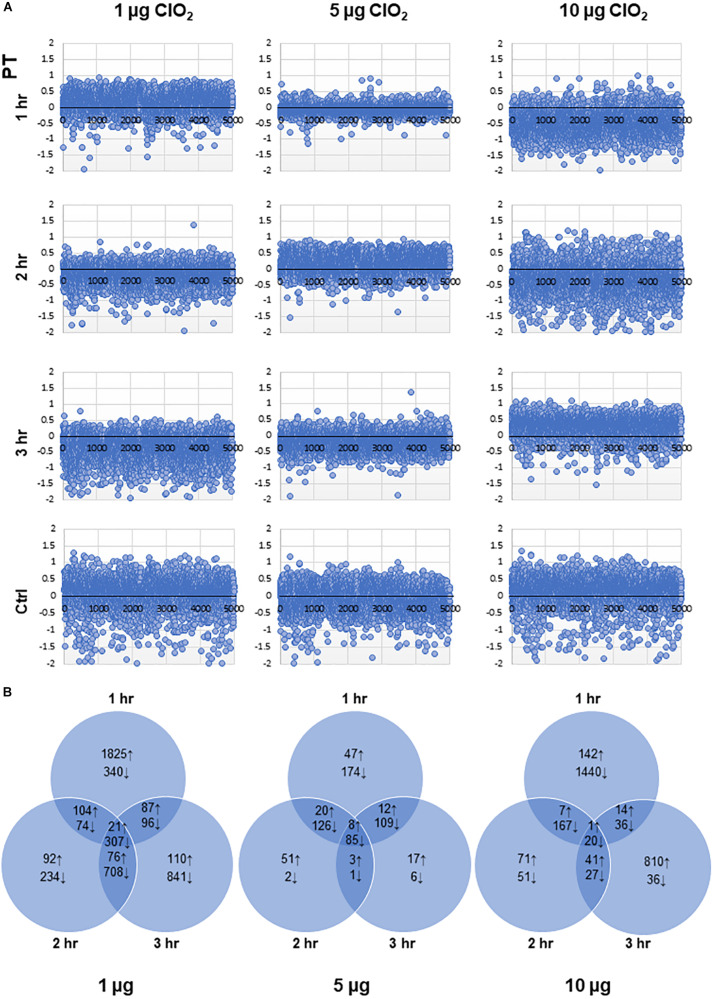
Dynamic changes in the transcriptomes of *E. coli* under different doses of ClO_2_. **(A)** Scatter plots showing the propensity transformation (PT) for each transcriptome library. **(B)** Venn diagrams showing the total number of upregulated (↑) and downregulated (↓) genes according to whether they are specific to exposure time or shared across time points for each dose of ClO_2_.

The dynamic temporal transcriptome profiles of *E. coli* O157:H7 in response to ClO_2_ (1, 5, and 10 μg ClO_2_) were obtained by transforming the RNA-Seq datasets from normalized RPKM values to propensity values using our novel PT methodology. This method provided a robust means to examine relative changes in expression across the time-course experiments. PCC of the PT relative expression values were further applied to determine the significance of gene expression changes. The scatter plots shown in Figure 2A indicated that the bacterial transcriptome activity, expressed as PT values, was remarkably increased within 1 h of exposure to 1 μg ClO_2_. The most drastic gene expression changes occurred between 1 h and 2 h showing massive decrease in expression while gene expression profiles at 2 h and 3 h, were similar. However, exposure to 5 μg ClO_2_ led to dynamic changes over time with decreased expression of a large subset of genes at the first hour and the third hour, but with more genes showing increased expression at the second hour. In stark contrast, massive decreases in gene expression were observed at the first and second hour when the bacterial populations were exposed to 10 μg ClO_2_. This indicates that xenobiotic effects of higher ClO_2_ dose perturb the overall physiological status of *E. coli* during the initial 2 h. Surprisingly, the bacterial transcriptome configuration was remarkably increased at the third hour of exposure to 10 μg ClO_2_ (Figure 2A).

Our data revealed an overlapping set of 4,076 (81.6% of total *E. coli* O157:H7 genes) mRNA-encoding loci with significant changes in abundance (either upregulated or downregulated) at one or more time points at 1 μg, 5 μg, and 10 μg ClO_2_ (Additional file 1: Supplementary Table S1). The highest magnitude of differential expression occurred at 1 h under 1 μg of ClO_2_ with 2,037 genes upregulated and 817 genes downregulated (Figure 2B). Notably, of the 2,037 upregulated genes during the first hour at 1 μg of ClO_2_, a total of 1,094 were downregulated at either 2 h, 3 h or both (Additional file 1: Supplementary Table S1). In contrast, the transcriptome at 2 h and 3 h under 1 μg ClO_2_ revealed only a total of 293 and 294 genes with significant upregulation. A much larger subset of 1,323 and 1,952 genes showed downregulation, respectively, in each time-point (Figure 2B). When the bacteria were exposed to 5 μg ClO_2_, more genes were continuously suppressed over time (Figure 2B), implying continuous bacterial perturbation, decline, and killing, although the log-reduction data did not show significant decline at the first hour (Figure 1). Only 87, 82, and 40 genes were significantly upregulated while a much larger set of 494, 214, and 201 genes were significantly downregulated at 1 h, 2 h, and 3 h, respectively (Figure 2B). The highest magnitude of downregulation, 1,663 genes, occurred at the first hour of exposure to 10 μg ClO_2_ (Figure 2B). This trend indicates that rather than defense (which was evident during the first hour of exposure to 1 μg ClO_2_), more severe perturbation had taken place causing massive bacterial cell death in the initial first hour of exposure to 10 μg ClO_2_ correlating with the drastic change in log-reduction (Figure 1). Notably with longer exposure, a new burst of defense responses were triggered under this high selection pressure with a total of 866 genes being upregulated at the third hour of exposure to 10 μg of ClO_2_ (Figure 2B). Among the 866 upregulated genes, a total of 454 were uniquely upregulated at this time point (Additional file 1: Supplementary Table S1). A significant proportion of this subset of ‘*transiently upregulated*’ genes are associated with type-III secretion system (T3SS), biofilm formation, prophage induction, and two-component system (Additional file 1: Supplementary Table S1). The large proportion of upregulated genes under this stress condition was suggestive that the bacterial population was shifting, with possible adaptation and selection occurring under such level of stress and exposure time.

**FIGURE 3:**
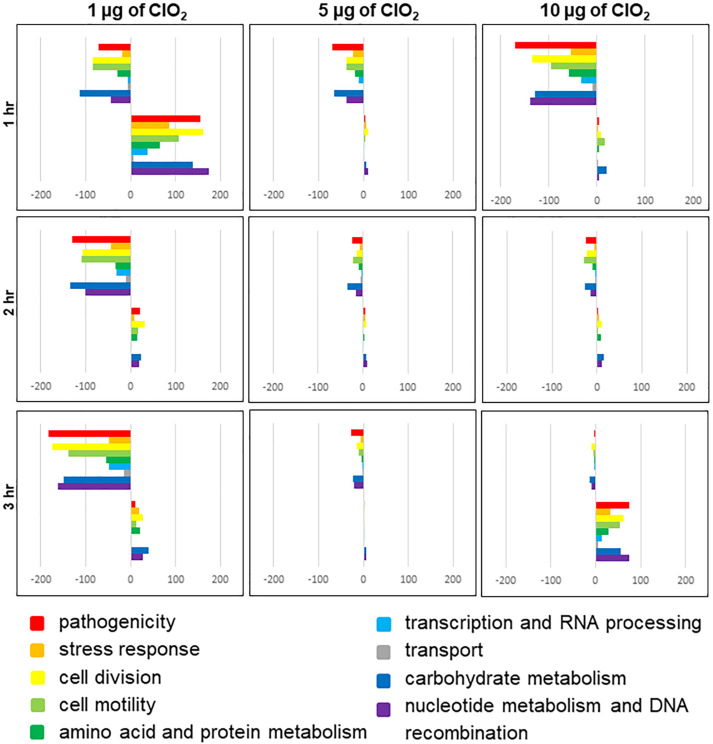
Functional categories of *E. coli* genes that are differentially regulated by ClO_2_ treatments after 1 h, 2 h, and 3 h exposure. Colored bars represent the number of regulated genes assigned to functional categories. Positive bars denote the number of upregulated genes. Negative bars denote number of downregulated genes. Genes assigned to ‘other category’ and ‘unknown’ are not included in this figure.

The *E. coli* genes observed to be differentially expressed in this study were predominantly associated with pathogenicity, stress response, cell division, cell motility, amino acid and protein metabolism, transcription and RNA processing, transport, carbohydrate metabolism, nucleotide metabolism, and genetic recombination (Figure 3 and Additional file 1: Supplementary Table S1). A large proportion of genes associated with pathogenesis and stress response were upregulated rather than downregulated during the first hour of exposure to 1 μg of ClO_2_ (Figure 3). However, a much larger proportion of pathogenesis and defense response genes were significantly downregulated once the bacteria were challenged with 1 μg of ClO_2_ for 2 h and 3 h, or 1 h under 5 and 10 μg of ClO_2_ (Figure 3). There appears to be a second burst of defense-associated genes that were upregulated during the third hour at 10 μg ClO_2_, with a large number of pathogenesis and defense response genes upregulated (Figure 3).

**FIGURE 4:**
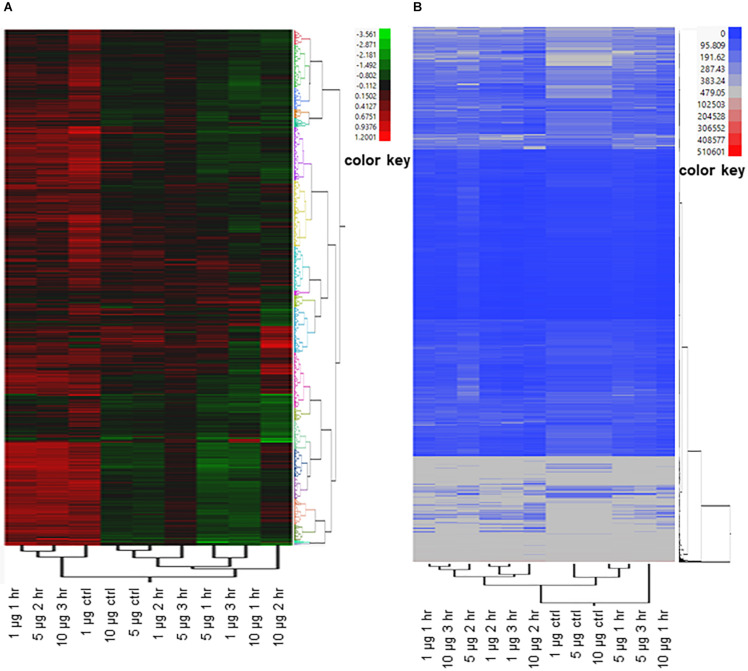
Two-way clustering of differentially expressed *E. coli* genes. Ward’s Hierarchical Clustering was performed to analyze propensity transformation (PT) values **(A)** and RPKM values **(B)** of *E. coli* genes in response to 1 μg, 5 μg, and 10 μg of ClO_2_ treatments after 1 h, 2 h, and 3 h. The number of clusters was set at 20 shown in Additional file 2: Supplementary Table S2, and clusters were color coded. Red indicates high expression and green (A)/blue (B) indicate low expression.

Many of the *E. coli* O157:H7 genes that were differentially expressed in response to exogenous ClO_2_ are either not well characterized or lacking informative functional annotation. To gain more insights into the possible functional significance of these genes to resistance or susceptibility to xenobiotic effects, two-way hierarchical clustering was performed by plotting both PT values and RPKM values, which formed a total of 20 distinct groups for each type of data (Figure 4 and Additional file 1: Supplementary Table S1). When the PT values were clustered by treatment, the ‘1 μg ClO_2_ × 1 h,’ ‘5 μg ClO_2_ × 2 h,’ and ’10 μg ClO_2_ × 3 h’ transcriptomes clustered together as the first major clade. The ‘10 μg ClO_2_ × 1 h’ and ‘10 μg ClO_2_ × 2 h’ transcriptomes clustered with the ‘1 μg ClO_2_ × 3 h’ transcriptome (Figure 4A). When the RPKM values were clustered by treatment, the ‘1 μg ClO_2_ × 1 h,’ ‘5 μg ClO_2_ × 2 h,’ and ‘10 μg ClO_2_ × 3 h’ transcriptomes also clustered together as the first major clade (Figure 4B). However, with the RPKM values, the ‘1 μg ClO_2_ × 2 h’ and ‘1 μg ClO_2_ × 3 h’ transcriptomes clustered with the ‘10 μg ClO_2_ × 2 h’ transcriptome (Figure 4B).

### Expression Changes in Pathogenicity and Stress Response Genes

**FIGURE 5:**
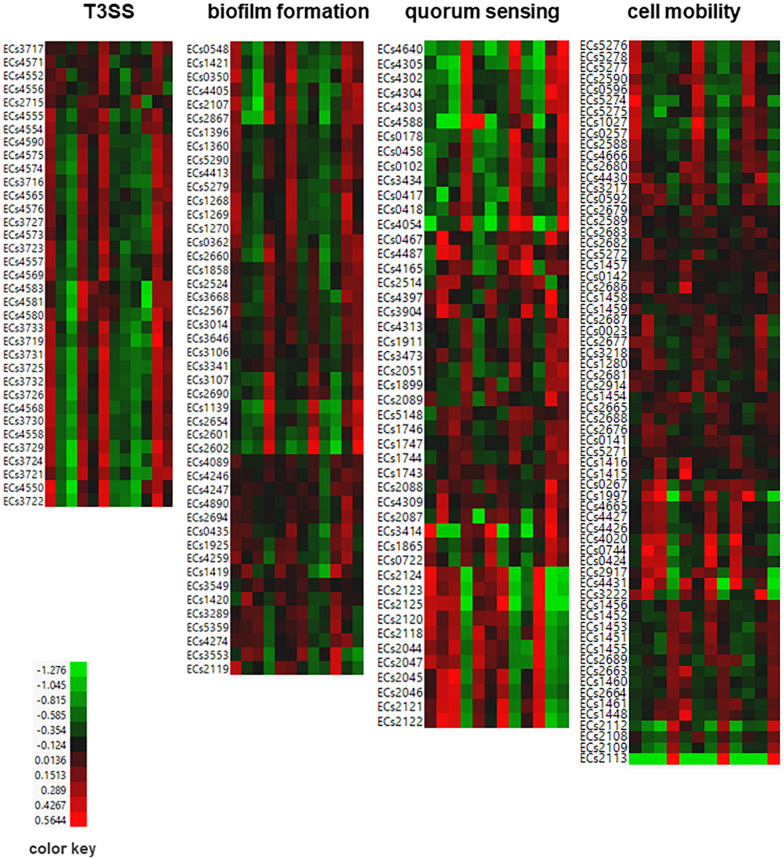
Heat map of differentially expressed *E. coli* genes associated with T3SS, biofilm formation, quorum sensing, and cell mobility. Propensity values of *E. coli* genes in response to 1 μg, 5 μg, and 10 μg of ClO_2_ after 1 h, 2 h, and 3 h were plotted. Red indicates high expression and green indicates low expression.

The bacterial T3SS are multi-protein complex channels that can inject effectors into host cells and promote bacterial attachment on host surface (McAteer et al., 2018). In this study, we examined the expression profiles of T3SS genes and found 25 and 13 of them to be significantly upregulated during the first hour of 1 μg ClO_2_ exposure, and during the third hour of 10 μg ClO_2_ exposure, respectively. This included the *EspF* (ECs4550) gene (Figure 5 and Additional file 1: Supplementary Table S1) which encodes the anchor structure protein of T3SS (Mainil, 2013). None of these T3SS genes were downregulated under these conditions (Figure 5 and Additional file 1: Supplementary Table S1). Intriguingly, a set of T3SS genes were downregulated at both 2 and 3 h of 1 μg ClO_2_ exposure and also at 1 h of 10 μg ClO_2_ exposure (Figure 5 and Additional file 1: Supplementary Table S1). However, only two of these T3SS genes were differentially expressed in response to 5 μg ClO_2_ *t* showing downregulation at 1 h and upregulation at 2 h (Figure 5 and Additional file 1: Supplementary Table S1). During the maximum growth reduction phases (2 h of 1 μg ClO_2_ exposure and 1 h of 10 μg ClO_2_ exposure, Figure 1), it is likely that the reduced ability of T3SS to secrete virulence factors was a consequence of reduction in bacterial ability to attach to the surface tissues of the non-host tomato. Moreover, under exposure to 1 μg ClO_2_, two Shiga toxin 2 (Stx2) (ECs1205, ECs1206) were significantly enhanced at 1 h and 2 h. Two Shiga toxin 1 (Stx1) (ECs2974, ECs2973) were dramatically attenuated at 2 h and 3 h (Additional file 1: Supplementary Table S1). These results indicate that the xenobiotic effects of ClO_2_ compromise pathogenicity mechanisms including adhesion ability, and these effects become particularly evident during the maximum growth reduction phases.

A total of 46 biofilm-associated genes appeared to be involved in defenses against the xenobiotic effects of ClO_2_ including several adhesion genes that were specifically upregulated during the first hour when defense and survival mechanisms were activated but downregulated at 2 h and 3 h at 1 μg ClO_2_ (Figure 5 and Additional file 1: Supplementary Table S1). The data also revealed an integration host factor (IHF) gene (ECs0995) functioning as negative regulator of flhDC and flagella (Yona-Nadler et al., 2003), which were downregulated at 1 h but upregulated at 3 h under 1 μg ClO_2_ exposure (Additional file 1: Supplementary Table S1). This suggests that pathogenicity genes were enhanced at the first hour of exposure but were suppressed at the third hour. Of these differentially expressed biofilm formation genes, 18 were downregulated with exposure to 10 μg ClO_2_ at 1 h, and 9 of these genes showed significantly increased expression at the third hour (Figure 5 and Additional file 1: Supplementary Table S1). In contrast, only two and seven biofilm formation-associated genes were upregulated and downregulated in response to 5 μg ClO_2_ treatment at 1 h, respectively (Figure 5 and Additional file 1: Supplementary Table S1).

Quorum sensing is the mechanism that bacteria cells employ in cell-to-cell communication for detecting xenobiotics, monitoring cell population density, and translating the extracellular signals to intercellular processes and subsequent gene expression (Ng and Bassler, 2009). Our data revealed a total of 47 quorum sensing associated genes to be induced by ClO_2_ exposure (Figure 5 and Additional file 1: Supplementary Table S1). Our data also showed a set of quorum sensing genes that were upregulated during the first hour of exposure to 1 μg ClO_2_ but were downregulated at both 2 and 3 h, including the signal recognition particle protein Ffh (ECs3473) (Figure 5 and Additional file 1: Supplementary Table S1). In addition, expression of the quorum sensing gene Ler protein (ECs4588) known to trigger other genes in the same pathogenicity island (McDaniel and Kaper, 1997; Mellies et al., 2002) was downregulated at 2 h and 3 h at 1 μg ClO_2_. When the bacteria were exposed to higher dosages of ClO_2_, several quorum sensing genes showed enhanced expression during exposure to 5 μg ClO_2_ but decreased expression during exposure to 10 μg ClO_2_, with a few showing dramatically increased expression to the xenobiotic effects of 10 μg ClO_2_ at the third hour (Figure 5 and Additional file 1: Supplementary Table S1).

Bacterial extracellular structures such as flagella, fimbriae, curli, and pili play important roles in pathogenicity, cell mobility, and biofilm formation (Mainil, 2013). Our data revealed 66 cell motility-associated genes to be differentially expressed in response to exogenous ClO_2_ (Figure 5 and Additional file 1: Supplementary Table S1). For instance, 36 flagellar, fimbriae, curli, and/or pili-associated genes were coordinately upregulated at 1 h in response to 1 μg ClO_2_, and then downregulated subsequently. Moreover, FliR (ECs2689) and a putative type 1 fimbrial protein precursor (ECs2113) were downregulated at all three time points at 5 μg ClO_2_. When the bacterial cells were exposed to 10 μg ClO_2_, a total of 34 cell mobility genes were downregulated at 1 h, but 16 of these genes were upregulated subsequently at 3 h (Figure 5 and Additional file 1: Supplementary Table S1).

The two-component system is a predominant mechanism by which bacterial cells respond to changing environments to maintain pathogenicity potential and fitness (Breland et al., 2017). A large number of *E. coli* two-component system associated genes showed different temporal induction profiles during exposure to exogenous ClO_2_ (Additional file 1: Supplementary Table S1). In response to 1 μg ClO_2_, while the expression of many two-component system-associated genes were enhanced during the onset of xenobiotic effects (1 h), seven were significantly downregulated at 3 h including *CpxR* (ECs4838), which is a critical player in the mammalian bactericidal peptidoglycan recognition protein (PGRP)-induced bacterial growth reduction (Kashyap et al., 2011, 2017), and *CpxA* (ECs4837) and *PhoQ* (ECs1601) genes encoding acid-responsive sensor kinases known to be required for the expression of virulence in *Salmonellae* (Prost et al., 2007), and which were also downregulated at 2 h (Additional file 1: Supplementary Table S1). When the bacterial cells were exposed to 5 μg ClO_2_, 14 genes associated with the two-component system were downregulated at one or more time point(s) including *CpxA* (ECs4837), *CpxR* (ECs4838), and *PhoQ* (ECs1601) (Additional file 1: Supplementary Table S1). While the bacteria cells are exposed to 10 μg ClO_2_, the expression of many two-component system-associated genes were suppressed at 1 h and subsequently enhanced with expression peaks occurring during the third hour (Additional file 1: Supplementary Table S1).

*E. coli* general stress response genes were shown to be induced in response to the ClO_2_-mediated perturbations. Phosphotransferase system HPr enzyme (ECs4354), a global regulator of energy metabolism (Rodionova et al., 2017), was upregulated at 1 h and downregulated at 2 h in response to 1 μg ClO_2_. However, at 10 μg ClO_2,_ the phosphotransferase system HPr protein was downregulated at 1 h but upregulated at 3 h (Additional file 1: Supplementary Table S1). A significant decrease in the expression of several SOS response genes, thiol stress-associated genes, putative tellurium resistance genes, lipopolysaccharide-associated genes, and chemotaxis-associated genes occurred during the first hour at 10 μg ClO_2_, then showed significant increase at the third hour (Additional file 1: Supplementary Table S1). However, a few heat shock protein (HSPs) genes were consistently inhibited during 5 μg ClO_2_ exposure, but several HSPs were induced at the first hour at 10 μg ClO_2_ (Additional file 1: Supplementary Table S1). In addition, we observed a large number of antibiotic biosynthesis and prophage associated genes to be differentially expressed in response to ClO_2_ exposure, indicating that the ClO_2_ induced defense response could be associated with antibiotic resistance and phage induction (Additional file 1: Supplementary Table S1).

In *E. coli*, transcriptional regulatory genes such as sigma-E factors are key players in biofilm formation and pathogenicity (Redford and Welch, 2006; Wang et al., 2017; Yang et al., 2018). In this study, we observed a set of transcriptional regulation-associated genes to be differentially expressed during exposure to ClO_2_ including three sigma-E regulatory proteins (ECs3436, ECs3437, ECs3438), which were upregulated specifically 1 h after 1 μg exogenous ClO_2_, and subsequently downregulated at later time points. These genes were upregulated during 3 h exposure to 10 μg ClO_2_ (Additional file 1: Supplementary Table S1). In addition, our data indicated that ribosome genes were suppressed by the xenobiotic effects of 5 μg ClO_2_ (Additional file 1: Supplementary Table S1). However, a few ribosome genes were upregulated at both 1 h and 3 h at 10 μg ClO_2_ (Additional file 1: Supplementary Table S1), suggesting that these two dosages of ClO_2_ induced very different defense responses in *E. coli*.

### Impact of ClO_2_ Xenobiotic Effects on Global Transcriptional Regulatory Networks

**FIGURE 6:**
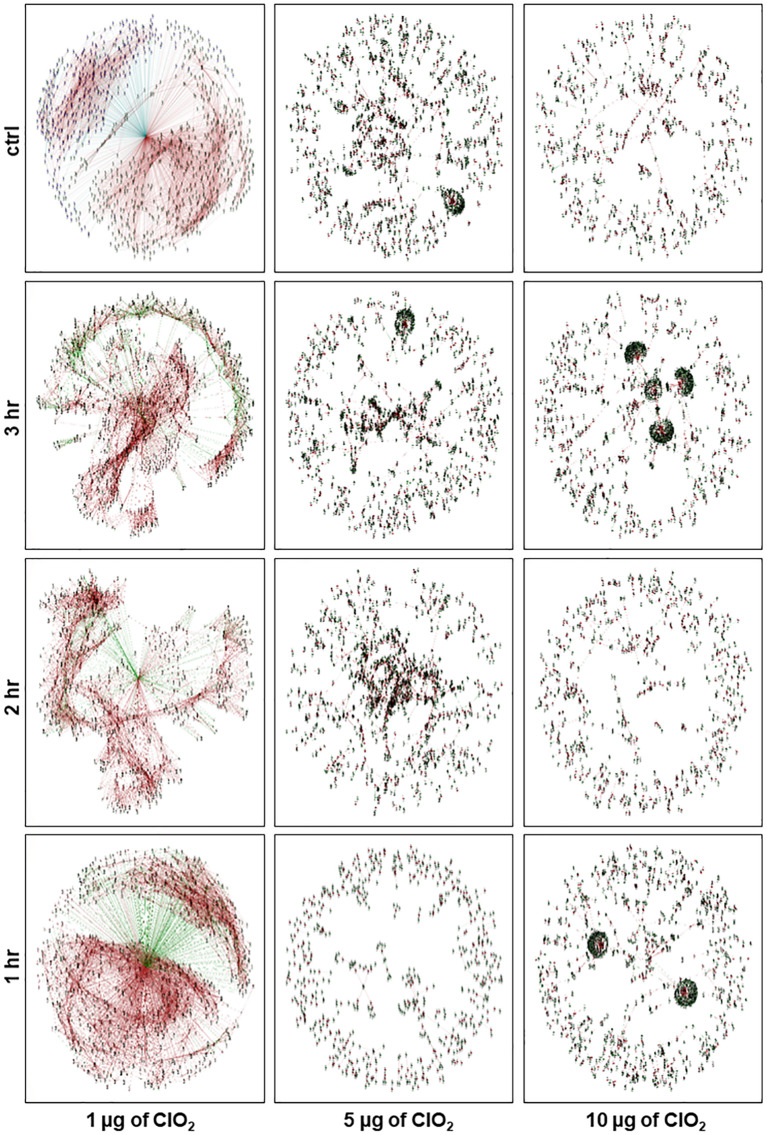
Network view of the constructed *E. coli* gene co-expression networks at control condition and after 1 h, 2 h, and 3 h of exposure to ClO_2_. Each node represents a gene and each line denotes the gene expression correlation between the two nodes. Green node denotes upregulated genes, blue node denotes downregulated genes, brown line denotes positively correlated by Pearson Correlation Coefficient (PCC), and green line denotes negatively correlated by PCC.

In order to better understand the global gene co-expression changes in *E. coli* during ClO_2_ exposure, we have reconstructed the transcriptional regulatory networks under different conditions. With *E. coli* cells being perturbed by 1 μg ClO_2_ during the first hour, a putative endopeptidase (ECs2739) was clearly located at the center of the global network (Figure 6). It is apparent that two large clusters of genes were coordinately regulated in either positive or negative manner by the putative endopeptidase gene that serves as the primary or central hub. A total of 1,575 genes were positively regulated by the putative endopeptidase, including several T3SS genes (ECs3730; ECs3731; ECs3732; ECs3733; ECs3716) (Figure 6 and Additional file 2: Supplementary Table S2).

In contrast, a total of 1,214 genes were negatively regulated by the putative endopeptidase, including those associated with pathogenicity, stress response, transcriptional regulation, and 22 ribosome-associated proteins (Figure 6 and Additional file 2: Supplementary Table S2). Other genes serving as secondary hubs during the defense and survival phase were further investigated by intra-module node connectivity including several prophage genes such as a putative terminase small subunit (ECs1970, ECs2252) and putative endolysin (ECs1213, ECs2968) that co-clustered positively with the putative endopeptidase central hub (Figure 6 and Additional file 2: Supplementary Table S2). Secondary network hubs that were downregulated by the putative endopeptidase included the *CpxR* (ECs4838) and *AtpB* (ECs4680) genes (Figure 6 and Additional file 2: Supplementary Table S2). Overall, the ClO_2_ response transcriptome during the initial treatment phase appeared to involve highly organized networks of co-upregulated and co-downregulated genes indicating that the bacterial defense systems were actively elicited against the non-lethal levels of xenobiotic stress (Figure 1).

Although the same putative endopeptidase gene occupied the core of the associated networks during the second hour at 1 μg ClO_2_, much fewer genes were positively regulated with 164 and 108 genes being positively and negatively regulated, respectively (Figure 6 and Additional file 2: Supplementary Table S2). Of the 164 positively regulated genes, five are pathogenicity-associated including SfmH protein (ECs0595). Of the 108 negatively regulated genes, only one (trp operon leader peptide, ECs1837) was associated with pathogenicity. During this phase, the network is dispersed with sub-clusters scattered around the center without forming large and well-organized clusters. Several genes acting as additional hubs were observed in this network, i.e., FliD protein (ECs2663), Ler protein (ECs4588), and MotB protein (ECs2599), all of which were negatively regulated by the central hub putative endopeptidase.

During the third hour at 1 μg ClO_2_, the putative endopeptidase still appeared in the center of the larger network. However, the network is even more disorganized compared with the 1 and 2 h networks with 222 and 71 genes being positively and negatively regulated, respectively (Figure 6 and Additional file 2: Supplementary Table S2). A total of 33 pathogenicity and stress-associated genes were positively regulated by this core gene at this phase, such as *OmpC*, *ArcA*, and *RpoS*. Only three stress response genes (cold shock protein, ECs0662; methyl-accepting chemotaxis protein I, ECs5315; aminomethyltransferase, ECs3776) were negatively regulated by the central hub gene with no pathogenicity genes being negatively regulated. At this phase, a large group of downregulated genes formed a cluster on one side of the network with putative endolysin (ECs1612) serving as one of the hubs. This gene was positively regulated by the putative endopeptidase while positively regulating another subset of genes including *MokW* (ECs2198). On the other side of the network were small sub-clusters distinct from the center. The upregulated superoxide dismutase (ECs2365) is one of the hub genes which was positively regulated by the putative endopeptidase, and appeared to be positively regulating a dihydrodipicolinate reductase (ECs0034) and proline dehydrogenase (ECs1260) genes.

During exposure to 5 μg ClO_2_, a few small clusters were clearly located at the center of the global network at 1 h (Figure 6). At the second and third hours, large clusters of genes were coordinately regulated in either positive or negative manner without an apparent central hub (Figure 6 and Additional file 2: Supplementary Table S2). However, genes serving as secondary hubs were observed, including a large cluster with lipoprotein Rz1 precursor (ECs1624) as a secondary hub at the third hour (Figure 6).

During exposure to 10 μg ClO_2_, a few large secondary network clusters were shown at the first and third hours of exposure. However, the second hour network was more disorganized (Figure 6). For instance, four large clusters with clear central hubs were detected at the third hour including the recombinase recT protein (ECs1933), putative endopeptidase (ECs2184), hypothetical protein (ECs1951) and hypothetical protein (ECs1949) (Figure 6). These results indicated that the second hour of xenobiotic effects by 10 μg ClO_2_ were the point when possible adaptation process began (Figure 6). The novel regulatory network formed at 3 h suggested that potential adaptive responses associated with prolonged exposure under high dose of ClO_2_ was triggered and clearly distinct from the events that happened at earlier stages.

Overall, both the 5 μg and 10 μg ClO_2_ regulatory networks are not well-organized. This suggests that the bacteria cells employed different mechanisms in response to higher doses of ClO_2_ xenobiosis compared with 1 μg ClO_2_. The disorganized configuration of the networks during these phases indicated that bacterial viability was in a severely attenuated status, and this was confirmed by the growth reduction assays (Figure 1).

### Regulatory Modules During the Entire Duration of Xenobiotic Effects

**FIGURE 7:**
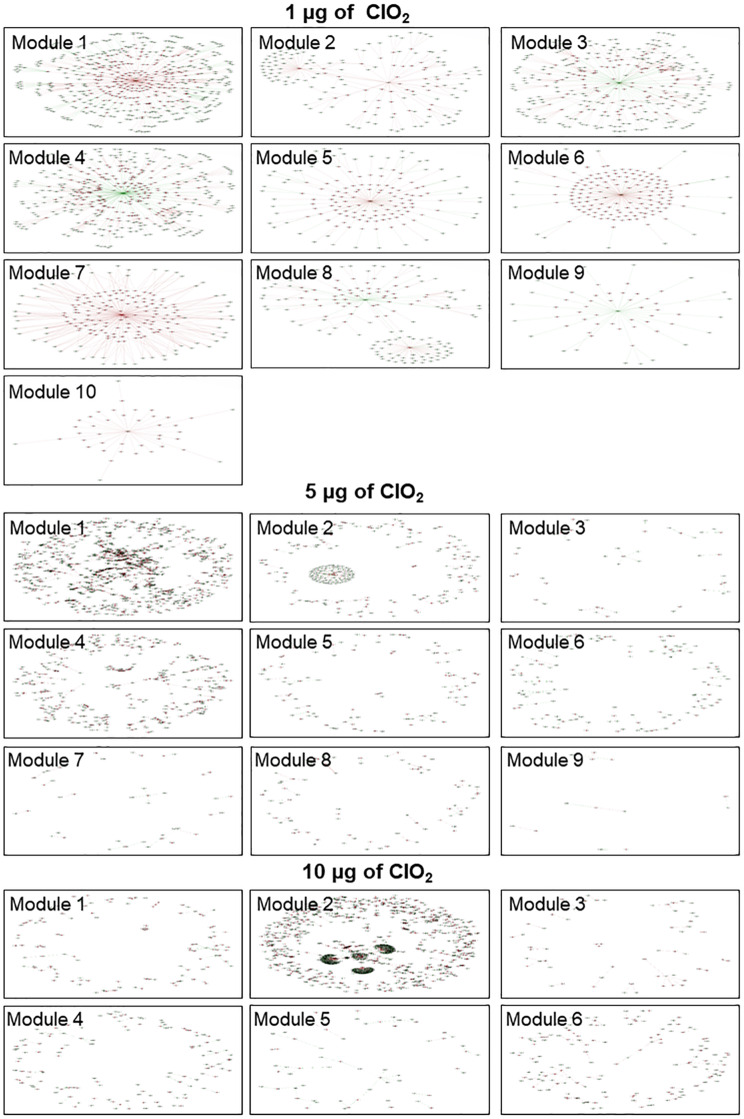
Network view of the *E. coli* gene co-expression modules. Nine (9) and six (6) modules were presented for 1, 5, and 10 μg ClO_2_ per gram of ripe fruits, respectively. Each node represents a gene and each line denotes the gene expression correlation between the two nodes. Red node denotes genes within the module, green node denotes genes not in the module but co-expressed with the genes within the module. Brown line denotes positive correlation and green line denotes negative correlation.

A total of ten (10), nine (9), and six (6) modules of highly correlated (positively co-expressed or negatively co-expressed) genes were obtained by integrative analysis of the entire duration of transcriptional changes under 1, 5, and 10 μg ClO_2_, respectively (Figure 7 and Additional file 3: Supplementary Table S3). The modules imply the temporal and sequential nature of cellular events and processes in response to each dose of ClO_2_. Analysis of the potential biological significance of these modules based on functional enrichment suggested their association with mechanisms of pathogenicity and stress response, prophage induction, sugar metabolism, and transcriptional regulation (Table 1).

**TABLE 1:** Gene functional enrichment of *E. coli* expression regulatory modules during the exposure to 1 μg, 5 μg, and 10 μg ClO_2_ per gram of ripe fruits.

**Module**	**Expression pattern**			**No. of genes**	**Functional enrichment**
	**Under 1 μ g ClO_2_**				
	**1 h**	**2 h**	**3 h**		

1	Up	Up	Up	185	Stress (biofilm formation/two-component/prophage)
2	Down	Down	Down	37	Prophage (endolysin/holin)
3	Down	/*	/	92	Stress (flagellar/chemotaxis)
4	Down	/	/	164	Stress (antibiotics)
5	Up	/	Up	96	Sugar (xylose/pentose)/stress (two-component/quorum sensing)
6	Up	UP	Down	135	Stress (SOS/antibiotics)
7	Up	Down	Down	352	Prophage/stress/sugar (fructose)
8	Down	Down	Up	53	Sugar (galactose)
9	Down	Up	/	40	Ribosome
10	Up	Down	Down	36	Pyrimidine

	**Under 5 μg ClO_2_**				

1	/*	Up	Down	508	Stress (prophage/T3SS)/sugar metabolism (fructose)/TF/transport
2	Down	/	Up	148	Stress (antibiotics)/ribosome
3	Up	down	/	92	Stress (prophage)/sugar metabolism
4	Down	/	/	201	Stress (antibiotics)/ribosome/transport
5	Up	/	Up	134	Stress (antibiotics/two-component)
6	Up	/	/	81	Stress (quorum sensing)/sugar metabolism
7	/	/	Up	67	Stress (antibiotics/two-component)
8	Down	Up	Down	50	Stress (antibiotics/tellurium resistance)
9	/	/	Down	37	Stress (chemotaxis/prophage)

	**Under 10 μg ClO_2_**				

1	/*	Up	Down	226	Stress (antibiotics/quorum sensing/two-component)
2	Down	/	Up	527	Stress (antibiotics/prophage/two-component)/TF/transport
3	Down	Down	Up	85	Stress (antibiotics/CAMP resistance)
4	Up	Down	Up	307	Stress (cold & heat shock protein/two-component)/TF
5	Up	Up	Down	58	Stress/TF
6	Down	Up	Up	83	Stress (prophage)

*Non-significant expression changes were indicated as ‘/’.

During exposure to 1 μg ClO_2_, most of the genes in each module were regulated by the putative endopeptidase except for Module-2 and Module-8 (Figure 7). In Module-2, an ABC transporter for maltose (ECs5081) appeared to function as a hub, regulating a number of genes associated with T3SS (i.e., SepL, ECs4557) (Figure 7). In Module-8, lipoprotein Rz1 precursor (ECs1624) is another obvious hub regulating another set of T3SS-associated genes such as *SepD* (ECs4574) and *CesD* (ECs4576) (Figure 7). While the bacteria cells were exposed to 5 μg ClO_2_ and 10 μg ClO_2_, most of the transcriptional regulatory modules are not well organized with genes distributed around the networks except for Module 2 in the 5 μg ClO_2_ treatment and Module-2 of the 10 μg ClO_2_ treatment, which showed clear sub-clusters (Figure 7). A lipoprotein Rz1 precursor (ECs1624) hub was evident in Module-2 of the 5 μg ClO_2_ treatment (Figure 7). However, four large clusters were shown in Module 2 of the 10 μg ClO_2_ treatment, with lipoprotein Rz1 precursor (ECs1624), putative endopeptidase (ECs2184), hypothetical protein (ECs1949) and recombinase recT protein (ECs1933) acting as the hubs for each cluster (Figure 7). Notably, all the hub genes shown in the modules also served as hub genes in the global transcriptional regulatory networks, which were discussed in the previous section (Figures 6, 7).

## Discussion

Shiga toxin producing *E. coli* O157:H7 is among the most potent food borne pathogens with importance to public health concerns due to its strong pathogenicity. *E. coli* O157:H7 is notoriously flexible in terms of its adaptability to extreme environmental fluctuations due in part to its short life-cycle, unicellularity, and highly efficient genetic regulatory machineries (i.e., operon system) (Allen and Griffiths, 2012; Chekabab et al., 2014). A very low level of *E. coli* O157 (i.e., 20 and 700 cells) in the food supply is capable of causing new outbreaks (Tuttle et al., 1999). Viable but non-culturable (VBNC) populations of bacterial cells could provide an effective inoculum when resuscitated under optimal environmental conditions (Gullian-Klanian and Sanchez-Solis, 2018). Moreover, the resilience of newly emerged isolates that are products of adaptation to strong selection pressures, such as those imposed by chemical intervention strategies, are often overlooked.

Gaseous ClO_2_ is one of the xenobiotic agents used to reduce the level of viable *E. coli* inoculum on fresh produce such as tomato, blueberry, strawberry, lettuce, and spinach (Sun et al., 2012; Ray et al., 2013; Banach et al., 2018; Park and Kang, 2018). It has been shown that a high dosage with short-duration exposure is sufficient to effectively disinfect *E. coli*-contaminated tomato, cantaloupe, and strawberry (Trinetta et al., 2013). Despite the increasing number of reports on the development of novel and presumably potent methods for disinfecting *E. coli*-contaminated tomato including the use of ClO_2_ as xenobiotic agent, major gaps need to be addressed. These gaps are created by the lack of fundamental knowledge on the molecular and genetic mechanisms associated with bacterial defenses, adaptive responses to xenobiotic stress, optimal dosage, treatment duration, and fine-tuned effects. Another major concern is the rapid appearance of strains that are better adapted to disinfection treatments as a consequence of sub-optimal or supra-optimal dosage, exposure time, or their combinations.

In this study, we directly compared the transcriptional changes in bacterial populations surviving on tomato surface during temporal treatment with different doses of ClO_2_ as a xenobiotic agent. We found that the O157:H7 strain of *E. coli* investigated in this study responded to ClO_2_ exposure in dosage and time-dependent manners, causing gene expression changes associated with pathogenicity, stress response, cell motility, transcriptional regulation, primary metabolism, and transport (Figure 3). Pathogenicity genes involved in T3SS, biofilm formation, quorum sensing, and two-component system were triggered by ClO_2_ (Figure 5). Examples include the Stx2 and Stx1 located in the lambdoid bacteriophage genomes that can be induced by lysogenic strains (Allen and Griffiths, 2012; Mei et al., 2015) suggesting that this organism can easily induce these genes to trigger chemical resistance and virulence. Although the ability of a pathogen to produce toxin is not sufficient for resistance to a stress or cause disease, induction of these genes could confer fitness and pathogenicity when combined with the effects of other virulence genes. The Stx2 and the adhesin were considered to be the most important virulence factors in *E. coli* O157 (Law, 2001). In addition, results of this study suggested that the responses of bacterial cells to exogenous ClO_2_ included the induction of resistance to oxidative and thiol stress (Additional file 1: Supplementary Table S1). This suggests that bacterial cells could potentially be adapted to other oxidizers, such as NaClO, ozone (O_3_), and hydrogen peroxide (H_2_O_2_). We are currently investigating the transcriptional regulatory networks of *E. coli* O157:H7 in response to different levels of O_3_ exposure in order to compare the nature of its genetic responses to the responses to ClO_2_.

We consistently address the apparent upregulation of many *E. coli* genes during the first hour of exposure to 1 μg ClO_2_, but downregulation at either or both 2 h and 3 h, which is probably due to the relatively more active physiological status at early stage of ClO_2_ exposure hence a more robust defense response. The subsequent compromise in bacterial viability was clearly a consequence of the prolonged exposure to the xenobiotic effects, reaching the maximum threshold of defense potential, hence steady decline. The substantial similarity in the overall transcriptome profiles and the associated regulatory networks during 2 h and 3 h exposure compared to the changes occurring between 1 h and 2 h indicate that bacterial populations on the surface tissues of non-host tomato, while undergoing significant growth reduction, could potentially lead to selection pressure upon prolonged exposure that triggers adaptive responses. Notably, we also revealed a number of pathogenicity genes that were upregulated at 3 h during exposure to 1 μg ClO_2_, such as *OmpC, ArcA*, and *RpoS* (Figure 5 and Additional file 1: Supplementary Table S1). The two-component gene *OmpC* is positively regulated by *OmpR*, which encodes a hypo-osmotic response regulator protein activated by the upstream sensor kinase EnvZ (Forst et al., 1989; Cai and Inouye, 2002; Schwan, 2009). *ArcA* gene encodes a virulence factor that is essential for flagellar motility, chemotaxis, and proper metabolic function (Jiang et al., 2015). It also functions as a transcriptional regulator that modulates bacterial adaptation during transition from anoxic to aerobic conditions (Gunsalus and Park, 1994; Georgellis et al., 2001; Loui et al., 2009). The *RpoS* gene has been highlighted as the most important gene for mediating general stress response in *E. coli* (Battesti et al., 2011; Landini et al., 2013).

When the bacteria cells were exposed to 5 and 10 μg ClO_2_, it is likely that novel defense mechanisms distinct from the mechanisms at 1 μg ClO_2_ were triggered. This is likely due to adaptation and selection under high doses of xenobiotic exposure. Notably, the downregulation of *E. coli* genes at 1 h but upregulation at 3 h was consistently observed once the bacteria were exposed to 10 μg ClO_2_, including several important virulence factors, Stx2 (ECs1205), and a few adhesins (ECs0548, ECs1396, ECs1360, ECs0350). The substantial similarity in the transcriptional changes during 1 h and 2 h exposure to 10 μg ClO_2_, and the clear dissimilarity of the stress response during 3 h indicate that prolonged exposure could potentially lead to a selection pressure that possibly triggers adaptation. The apparent time-dependent gene expression patterns under both low and high doses of exogenous ClO_2_ suggested that toxicity effects, as well as genetic mechanisms, might be different under different doses of xenobiotic at various duration of exposure. Taken together, the results of the transcriptome analysis presented in this study indicate that novel defense mechanisms were triggered by 10 μg ClO_2_ at 3 h, and that these were likely triggered by potential adaptive responses to selection pressure under supra-optimal ClO_2_ exposure. These adaptive and selection effects may have occurred among a minority of the bacterial population that survived the incremental negative effects of xenobiotic exposure, hence creating VBNC. Such findings may be often overlooked in the food industry and are quite important in designing effective intervention protocols. The clustering of genes with known and unknown functions based on the timing and magnitude of their expression promises to aid in further understanding the *E. coli* response to chemical stresses, as well as providing a guide for the selection of candidate genes and pathways for further experimentation toward understanding the mechanism under which these affect bacterial behavior and responses to stress.

The transcriptional regulatory networks of *E. coli* O157:H7 have been extensively studied and aided by the available genome sequence resources (Perna et al., 2001; Wang et al., 2013; Santos-Zavaleta et al., 2018). In this study, we analyzed the transcriptional co-expression networks of *E. coli* O157:H7 surviving on the non-host tomato surface after exposure to different doses of ClO_2_. We reported that the transcriptional regulatory networks in response to 1 μg ClO_2_ are controlled by a single central hub (putative endopeptidase, ECs2739), which is likely to be associated with stress signaling, antibiotic binding and recognition, bacteriophage activity, and morphology determination (Meberg et al., 2004; Lood et al., 2017). However, when the bacterial populations were subjected to 5 μg and 10 μg ClO_2_, a few small clusters with distinct hubs were observed, including another putative endopeptidase (ECs2184). It is likely that the responses to ClO_2_ mimic the typical response to a biological invasion that often involves efforts to degrade foreign proteins by enhanced endopeptidase activities (Chang et al., 2012; Kashyap et al., 2017). In addition, the predominance of antibiotic biosynthetic genes and prophage induced genes is consistent with such observations. Our transcriptional regulatory networks further indicated that, when optimizing ClO_2_ treatment dosages and exposure time for intervention, potential adaptation needs to be taken into consideration.

Systematic reconstruction of the gene co-expression modules showed that genes associated with pathogenicity, stress response, prophage, sugar metabolism, nucleotide metabolism, transcriptional regulation, and transport play important roles in defense against ClO_2_ stress (Table 1). In *E. coli*, prophage induction is often coupled with enhanced virulence and increased tolerance to harsh environmental conditions (Fang et al., 2017; Li et al., 2018). Our data indicates that stress response and prophage gene induction are associated with the reallocation of energy resources from growth and reproduction to defense response during the first hour, but increased energy generation coupled with suppressed defense response was prominent during 2 h and 3 h of exposure to 1 μg ClO_2_. These were likely to be due to low metabolic activities concurrent with cell division arrest (Jozefczuk et al., 2010). However, stress response and prophage induction-associated genes were downregulated during the first hour of exposure but upregulated during 2 h and 3 h at 10 μg ClO_2_ (Table 1). Enhanced expression of prophage induction associated genes at later time points strongly indicated potential adaptation with prolonged exposure to 10 μg ClO_2_. However, during exposure to 5 μg ClO_2_, prophage induction gene expression modules were downregulated at 3 h (Table 1), suggesting that under this stress condition, adaptation is not likely induced.

Current opinions support that, when exposed to initial stimulation, bacterial populations with the ability to maintain their viability (VBNC) may survive better across different host or non-host environments than those that have not been subjected or primed to stress (Gunasekera et al., 2008; Zorraquino et al., 2017). In *E. coli*, stress conditions could induce acclimation, adaptation, selection, or even rare mutation events. The fitness changes induced by the environmental stimuli could lead to selection and population shift to build a novel inoculum tolerant to a broad spectrum of stress conditions. One notable example is that *E. coli* O157 was reported to be more resistant to acid once it was primed by heat stress (Wang and Doyle, 1998). In *E. coli*, whether ClO_2_ causes mutagenic effects and subsequently contributes to cross-protection is unknown. From a food safety perspective, novel and sustainable strategies such as introducing multiple or combinations of relatively mild chemical treatments (i.e., optimal cocktail) might be an attractive alternative to completely decimate the bacterial inocula without promoting adaptation or mutation.

Past efforts showed that various stress conditions induced changes in *E. coli* gene expression in both pure culture and on non-host environments such as fresh lettuce (Allen and Griffiths, 2012; Mei et al., 2017). In this study, we analyzed the dynamic co-expression networks of *E. coli* in response to different doses of ClO_2_ on non-host tomato surfaces, which could serve as a pre-exposure to another stressor causing either cross-protection or cross-vulnerability (Zorraquino et al., 2017). We found that the genetic regulatory network configuration of *E. coli* is very flexible under different doses of ClO_2_ over time. We are currently characterizing transcriptional changes of *E. coli* growing in pure culture and on tomato surface to answer these questions.

The current study proved the feasibility of using next generation sequencing technologies as a novel approach to uncover the temporal co-expression networks in *E. coli* induced by gaseous ClO_2_. We have established a new platform to analyze the molecular genetic mechanisms underlying *E. coli*-ClO_2_ interactions, which provided a powerful tool for future transcriptional profiling and gene discovery. The differentially expressed genes could serve as targets for the development of novel control strategies for foodborne pathogens. New chemicals and bio-control agents, i.e., non-toxic and non-pathogenic biocontrol bacterial strains or phage, can be considered. In addition, the manipulation of *E. coli* metabolic and signal transduction pathways associated with defense response to ClO_2_ could be an alternative strategy to re-wire the bacterial genetic networks, thereby reducing selective pressure and avoiding the emergence of chemical tolerant inoculum. The information generated in this study provides and important resource for future food safety and epidemiology research. These findings are important in the prevention of new outbreaks caused by foodborne pathogens.

**FIGURE 8:**
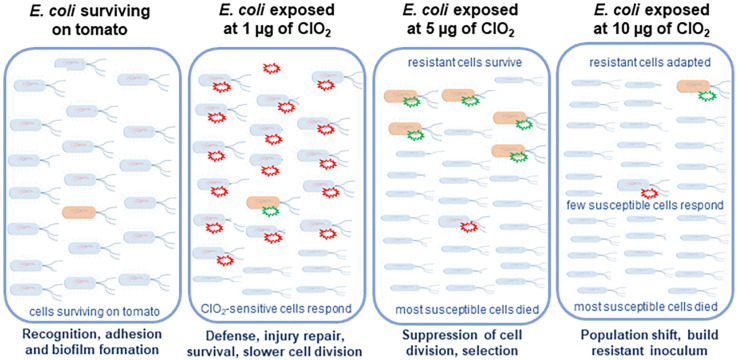
Hypothetical model of the sequential defenses of in *E. coli* against ClO_2_ on its non-host tomato environment. Xenobiotic effects of 1 μg of ClO_2_ resulted in the induction of pathogenesis and stress responses in the majority of the cells which were sensitive. However, 5 μg of ClO_2_ treatment induced massive cell death and selection of several tolerant cells. Under high dose of ClO_2_ treatment, which is 10 μg, with massive cell killing, resistant cells adapted to the environmental stress.

The present study serves as a proof of concept that the xenobiotic agent ClO_2_ could induce potential adaptation and selection in *E. coli* (Figure 8). We demonstrated how the transcriptional regulatory networks changed over time under different ClO_2_ doses. Such an experimental system could be used to evaluate the impacts of different intervention strategies in the food industry at the molecular genetic level to eliminate bacterial pathogens surviving on fresh produce.

## Data Availability Statement

The sequence files are available at NCBI SRA under accession number PRJNA516233 PRJNA516233 (https://www.ncbi.nlm.nih.gov/bioproject/?term=PRJNA516233).

## Author Contributions

BD conceptualized and supervised the whole study in collaboration with VW, interpreted the data and co-wrote the manuscript with XS. MS, DB, and VW performed all the microbial works and chemical treatments at USDA-ARS-WRRC. MS and DB also prepared all samples for the RNA-Seq libraries. AK designed the RNA-Seq experiments and assembled the Illumina sequence reads. XS and MS performed the biological interrogation and analysis of the RNA-Seq data. XS and NK performed all bio-computing works, statistical analyses, and genetic network modeling.

## Conflict of Interest

The authors declare that the research was conducted in the absence of any commercial or financial relationships that could be construed as a potential conflict of interest.

## Footnotes

1 www.genome.jp
