# Supplementary material for: Xenobiotic Effects of Chlorine Dioxide to Escherichia coli O157:H7 on Non-host Tomato Environment Revealed by Transcriptional Network Modeling: Implications to Adaptation and Selection
Source: Front Microbiol. 2020 Jun 3;11:1122. doi: 10.3389/fmicb.2020.01122 (PMC7286201; doi:10.3389/fmicb.2020.01122)
Supplement: Supplementary file 4 [file Data_Sheet_1.docx]

**Additional files**

**Additional file 1: Table S1** Significance (up or down-regulated based on Pearson Correlation Coefficient), propensity and RPKM of *E. coli* genes during treatment of 1, 5, and 10 µg of ClO_2_ after 1, 2, and 3 hour (hr). The clusters in columns AN and AQ were shown in Fig. 4. (XLSX 1,261 KB)

**Additional file 2: Table S2 Sheet 1** Propensity of *E. coli* genes selected for 1 µg ClO_2_ network analysis. The 250 most highly expressed and 250 most lowly expressed genes from each treatment [control, 1, 2, and 3 hour (hr)]. Label numbers from 0 to 19 denote genes from most lowly expressed to most highly expressed. Only those fall into either label 0 or 19 at least in one treatment were selected. Genes differentially expressed based on Pearson Correlation Coefficient (PCC) were shown in columns L-N. **Sheet 2** Propensity of *E. coli* genes selected for 5 µg ClO_2_ network analysis. The 250 most highly expressed and 250 most lowly expressed genes from each treatment [control, 1, 2 and 3 hour (hr)]. Label numbers from 0 to 19 denote genes from most lowly expressed to most highly expressed. Only those fall into either label 0 or 19 at least in one treatment were selected. Genes differentially expressed based on PCC were shown in columns L-N. **Sheet 3** Propensity of *E. coli* genes selected for 10 µg ClO_2_ network analysis. The 250 most highly expressed and 250 most lowly expressed genes from each treatment [control, 1, 2 and 3 hour (hr)]. Label numbers from 0 to 19 denote genes from most lowly expressed to most highly expressed. Only those fall into either label 0 or 19 at least in one treatment were selected. Genes differentially expressed based on Pearson Correlation Coefficient were shown in columns L-N. **Sheet 4** Co-expression network of *E. coli* genes for 1 µg ClO_2_ at 1 hr. 'color': 'brown' demotes positively correlated by PCC; 'color': 'green' demotes negatively correlated by PCC. **Sheet 5** Co-expression network of *E .coli* genes for 1 µg ClO_2_ at 2 hr. 'color': 'brown' demotes positively correlated by PCC; 'color': 'green' demotes negatively correlated by PCC. **Sheet 6** Co-expression network of *E. coli* genes for 1 µg ClO_2_ at 3 hr. 'color': 'brown' demotes positively correlated by PCC; 'color': 'green' demotes negatively correlated by PCC. **Sheet 7** Co-expression network of *E. coli* genes for 1 µg ClO_2_ control. 'color': 'brown' demotes positively correlated by PCC; 'color': 'green' demotes negatively correlated by PCC. **Sheet 8** Co-expression network of *E. coli* genes for 5 µg ClO_2_ at 1 hr. 'color': 'brown' demotes positively correlated by PCC; 'color': 'green' demotes negatively correlated by PCC. **Sheet 9** Co-expression network of *E. coli* genes for 5 µg ClO_2_ at 2 hr. 'color': 'brown' demotes positively correlated by PCC; 'color': 'green' demotes negatively correlated by PCC. **Sheet 10** Co-expression network of *E. coli* genes for 5 µg ClO_2_ at 3 hr. 'color': 'brown' demotes positively correlated by PCC; 'color': 'green' demotes negatively correlated by PCC. **Sheet 11** Co-expression network of *E. coli* genes for 5 µg ClO_2_ control. 'color': 'brown' demotes positively correlated by PCC; 'color': 'green' demotes negatively correlated by PCC. **Sheet 12** Co-expression network of *E. coli* genes for 10 µg ClO_2_ at 1 hr. 'color': 'brown' demotes positively correlated by PCC; 'color': 'green' demotes negatively correlated by PCC. **Sheet 13** Co-expression network of *E. coli* genes for 10 µg ClO_2_ at 2 hr. 'color': 'brown' demotes positively correlated by PCC; 'color': 'green' demotes negatively correlated by PCC. **Sheet 14** Co-expression network of *E. coli* genes for 10 µg ClO_2_ at 3 hr. 'color': 'brown' demotes positively correlated by PCC; 'color': 'green' demotes negatively correlated by PCC. **Sheet 15** Co-expression network of *E. coli* genes for 10 µg ClO_2_ control. 'color': 'brown' demotes positively correlated by PCC; 'color': 'green' demotes negatively correlated by PCC. (XLSX 1,651 KB)

**Additional file 3: Table S3 Sheet 1** Propensity and module of *E. coli* genes during treatment of 1 µg ClO_2_ after 1, 2, and 3 hour (hr). Co-expression network of each module was showing in Fig. 7. Different modules were separated by different colors for easy view. **Sheet 2** Propensity and module of *E. coli* genes during treatment of 5 µg ClO_2_ after 1, 2, and 3 hour (hr). Co-expression network of each module was showing in Fig. 7. Different modules were separated by different colors for easy view. **Sheet 3** Propensity and module of *E. coli* genes during treatment of 10 µg ClO_2_ after 1, 2, and 3 hour (hr). Co-expression network of each module was showing in Fig. 7. Different modules were separated by different colors for easy view. (XLSX 390 KB)
